# Evaluation of the prognostic potential of histopathological subtyping in high-grade serous ovarian carcinoma

**DOI:** 10.1007/s00428-024-03807-7

**Published:** 2024-04-16

**Authors:** Hein S. Zelisse, Robin A. Hwan, Marc J. van de Vijver, Frederike Dijk, Constantijne H. Mom, Gerrit K. J. Hooijer, Mignon D. J. M. van Gent, Malou L. H. Snijders

**Affiliations:** 1 Department of Pathology, Cancer Center Amsterdam, Amsterdam Reproduction & Development Research Institute, Amsterdam UMC, University of Amsterdam, Amsterdam, the Netherlands; 2 Department of Pathology, Amsterdam UMC, University of Amsterdam, Amsterdam, the Netherlands; 3 Department of Pathology, Cancer Center Amsterdam, Amsterdam UMC, University of Amsterdam, Amsterdam, the Netherlands; 4 Department of Gynaecologic Oncology, Centre for Gynaecologic Oncology Amsterdam, Cancer Center Amsterdam, Amsterdam UMC, University of Amsterdam, Amsterdam, the Netherlands

**Keywords:** Epithelial ovarian carcinoma, High-grade serous ovarian carcinoma, Subtypes, Prognosis

## Abstract

High-grade serous ovarian carcinoma (HGSOC) can be categorized into four gene expression-based subtypes, with supposedly distinct prognoses and treatment responses. Murakami et al. translated these gene expression-based subtypes into the histopathological mesenchymal, immunoreactive, solid and proliferative, and papilloglandular subtypes, showing differences in survival outcomes. Miyagawa et al. refined these criteria to improve the interobserver concordance. The current retrospective study evaluated the interobserver variability and the prognostic differences between the histopathologic subtypes using the criteria of both Murakami et al. and Miyagawa et al. in 208 HGSOC cases. The mesenchymal subtype was considered first, followed by the immunoreactive subtype. Non-conforming cases were categorized as solid and proliferative or papilloglandular. The mesenchymal subtype was identified in 122 patients (58.7%) for both criteria. Using the criteria of Murakami et al., 10 cases (4.8%) were immunoreactive, 26 (12.5%) solid and proliferative, and 50 (24%) papilloglandular, with a concordance rate of 62.5% (*κ* = 0.34, *p* < .001). Using the Miyagawa et al. criteria, 23 cases (11%) were immunoreactive, 20 (9.6%) solid and proliferative, and 43 (20.7%) papilloglandular. No survival differences were observed between the subtypes. The fair reproducibility of the histopathological subtype classification of HGSOC and the lack of survival differences among these subtypes indicate the need for further refinement of the criteria and exploration of their correlation with overall survival outcomes before clinical application.

**Supplementary Information:**

The online version contains supplementary material available at 10.1007/s00428-024-03807-7.

## Introduction

High-grade serous ovarian carcinoma (HGSOC) is the most common histotype of epithelial ovarian carcinoma (EOC), accounting for nearly 75% of all cases. With a 5-year overall survival (OS) rate of approximately 40%, it is also the most lethal histotype [1]. About 80% of HGSOC patients are diagnosed with advanced-stage disease, classified as stage III or IV according to the International Federation of Gynecology and Obstetrics (FIGO) criteria [2, 3]. Despite an initial high chemotherapy response rate of 85%, the 5-year OS rate of these patients with advanced-stage disease is below 30%, primarily due to the development of chemotherapy resistance [1, 2, 4–6]. In contrast, patients with early-stage disease (FIGO stages I and II) have a 5-year OS rate exceeding 70% [1, 3, 5].

Even when accounting for clinical characteristics such as age, outcome of debulking surgery, and FIGO stage, variations exist in chemotherapy responsiveness, recurrence rates, and OS rates [6–10]. Within HGSOC, four specific molecular subtypes were identified through microarray gene expression profiling and further delineated as the mesenchymal, immunoreactive, proliferative, and differentiated subtype [8, 9]. With a median OS of less than 30 months, the mesenchymal subtype had the poorest prognosis, whereas the immunoreactive subtype showed the best prognosis, with a median OS extending beyond 45 months [7, 10]. Moreover, retrospective studies showed varying treatment responses among these subtypes. For example, the proliferative subtype seemed to have the best response to carboplatin, while the mesenchymal subtype was particularly receptive to taxane-based chemotherapy [11]. Additionally, both the proliferative and mesenchymal subtypes appeared to have the greatest benefit of bevacizumab [12]. As such, these molecular subtypes may not only serve as potential prognostic indicators but also contribute to a more personalized treatment approach. However, the high costs, difficult sample quality assurance, and time-intensive nature of gene expression analysis limit routine clinical application.

To overcome this limitation, a histopathological approach for HGSOC subtyping has been proposed by Murakami et al. (2016) [13]. They introduced a classification system of four histological subtypes based on the initial molecular classification and observed significant differences in OS between these subtypes. Tumors with a destructive desmoplastic reaction and a mesenchymal transition or labyrinthine pattern were classified as mesenchymal, exhibiting the lowest OS rates. Tumors displaying a smooth invasive front and an immunoreactive pattern, defined as a median of more than 100 surrounding and 50 infiltrating lymphocytes from five visual fields at × 400 magnification, were labeled immunoreactive, showing the longest progression-free survival (PFS) and OS. The remaining tumors were categorized into the solid and proliferative subtype or the papilloglandular subtype, corresponding to the gene expression-based proliferative and differentiated subtypes, respectively. However, the authors reported that the reproducibility of the subtypes was considered suboptimal for clinical practice, with an average consistency rate of 74% between six observers and an average Cohen’s *κ* coefficient of 0.64 [13, 14].

In their next study, Miyagawa et al. (2023) observed a similar low concordance rate, with a Fleiss’ *κ* coefficient of only 0.348, when applying these criteria to whole slide images (WSI). Consequently, they further delineated the criteria for the mesenchymal, solid and proliferative, and papilloglandular subtypes. Moreover, given the challenges of observing cellular details on WSIs, they adapted the definition of the immunoreactive subtype to require at least 25% of the stromal area to be infiltrated by lymphocytes. These refined and adjusted criteria improved the Fleiss’ *κ* coefficient to 0.549, but differences in survival between the subtypes based on these criteria were not evaluated [14].

The objective of the current study was to assess the clinical utility of HGSOC histological subtypes within a cohort of patients with HGSOC. To achieve this, we examined the interobserver reliability to ascertain reproducibility and analyzed the prognostic variances in survival among histopathological subtypes of HGSOC, as defined by Murakami et al. (2016) and Miyagawa et al. (2023) [13, 14]. The findings of this study may contribute to a more personalized treatment approach for HGSOC by improving the prediction of prognosis and forming the basis of future research into treatment response differences between the subtypes.

## Materials and methods

### Study design and participant selection

The inclusion criteria of this single-center, retrospective cohort study were surgically treated patients with HGSOC, with the availability of hematoxylin and eosin-stained (H&E) sections or formalin-fixed paraffin-embedded tissue blocks from the primary ovarian tumor and clinical follow-up data of at least 3 years. Patients with a concurrent malignancy at the time of diagnosis were excluded.

Patients treated at Amsterdam University Medical Center (UMC), location Academic Medical Center (AMC), between 2011 and 2020, were identified based on the inclusion and exclusion criteria. The H&E sections from these patients were independently reviewed by two gynecological pathologists (MV and MS) to confirm the diagnosis of HGSOC. Any discordance between the pathologists was resolved through discussion. Upon confirmation, patients were included in the study.

### Histopathological evaluation

**Fig. 1:**
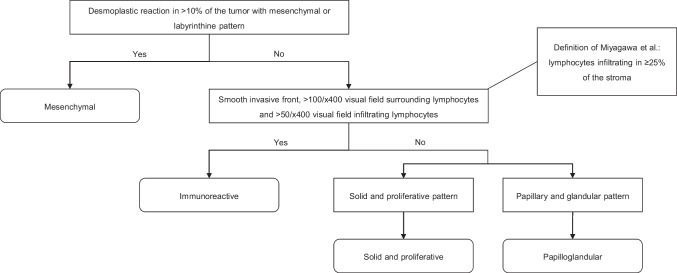
Classification criteria of Murakami et al., with the definition of Miyagawa et al. for the immunoreactive histotype incorporated

All cases were independently classified by two specialized gynecological pathologists (MV and MS). First, the cases were categorized according to the criteria of Murakami et al. (Fig. 1) [13]. However, for classifying the mesenchymal, solid and proliferative, and papilloglandular subtypes, we adhered to the refined criteria set by Miyagawa et al., which offered a more clearly outlined description while adhering to the fundamental definitions of Murakami et al. For the immunoreactive subtype, of which the definition was different between Murakami et al. and Miyagawa et al., we adhered to the original definition provided by Murakami et al. [13, 14].

Cases showing a desmoplastic reaction in > 10% of the tumor, accompanied by a mesenchymal or labyrinthine pattern, were identified as mesenchymal. The mesenchymal pattern was characterized by a destructive stromal reaction with spindle and isolated cells, while the labyrinthine pattern was identified by desmoplastic stroma with broad, compressed large papillae infiltration [13, 14]. If a tumor did not meet these criteria, the number of lymphocytes surrounding and infiltrating cancer nests was counted across five visual fields at × 400 magnification. Tumors showing a median of more than 100 surrounding and 50 infiltrating lymphocytes per visual field, along with a smooth invasive front, were labeled as immunoreactive [13]. Tumors not classified as mesenchymal or immunoreactive were categorized as solid and proliferative, or as papilloglandular, depending on which one occupied the greatest area of the tumor [13, 14]. Any classification discordance between MV and MS was resolved through joint review and discussion.

For whole slide images, Miyagawa et al. redefined the immunoreactive subtype to include non-mesenchymal cases with lymphocytes infiltrating 25% or more of the stromal area on the slide (Fig. 1). This percentage corresponds to the fraction of the stromal area covered by mononuclear immune cells within the invasive tumor borders, with necrotic areas excluded. If the distribution of lymphocytes was heterogeneous, an average percentage was used [14, 15]. We applied this revised definition to all cases initially jointly classified as immunoreactive, solid and proliferative, and papilloglandular according to the criteria of Murakami et al. Cases were independently reclassified, either as immunoreactive following the definition of Miyagawa et al. or as their original classification of solid and proliferative or papilloglandular. In case of classification discrepancies, consensus was reached through joint review and discussion.

### Statistical analysis

Differences in continuous variables were assessed using the one-way ANOVA test or the Kruskal–Wallis H test. Categorical variables were compared using the chi-square (χ2) test or Fisher’s exact test. The interobserver reproducibility of histopathological subtypes was assessed using Cohen’s *κ* coefficient. Both OS and PFS rates were calculated using the Kaplan–Meier method and presented as medians. PFS was defined as the duration from diagnosis to disease progression, recurrence, or the final follow-up moment. The log-rank test was used to assess differences in univariate survival outcomes. Multivariable Cox-regression analyses were conducted considering the subtypes, combined with age (below or older than median age), FIGO stage (I and II versus III and IV), and the outcome of surgery (presence or absence of macroscopic residual tumor) when significant in univariate survival analyses. A *p*-value below 0.05 was considered statistically significant. All statistical analyses were performed using SPSS version 28.0 (IBM Corp., Armonk, NY, USA) and GraphPad Prism, version 9.5 (GraphPad Software, San Diego, CA, USA).

## Results

### Clinicopathological characteristics

In total, 208 patients with HGSOC were included in the study (Table 1). The median age was 66 years, ranging from 35 to 85 years. A vast majority of the patients (93.3%) were diagnosed with FIGO stage III or IV. This high prevalence can likely be attributed to the cohort originating from Amsterdam UMC, a tertiary referral hospital. Patients diagnosed with early-stage ovarian carcinoma during adnexal extirpation at non-tertiary hospitals are routinely referred to our institution for subsequent staging surgery. However, in many cases, no residual tumor is detected during this procedure. Consequently, these patients could not be included in this study due to the absence of available tumor material within the Amsterdam UMC.

Nearly all patients (97.6%) underwent debulking surgery. Of these patients, 27.1% underwent primary and 72.9% underwent interval debulking surgery. Complete eradication of macroscopic tumors was achieved in 60.6% of these patients. Optimal debulking (< 1 cm macroscopic residual tumor) was achieved in 31%, and incomplete debulking (≥ 1 cm macroscopic residual tumor) in 8.4%. The mean number of chemotherapy cycles received was six. The median PFS was 17.8 months, and the median OS was 41.5 months.

**Table 1:** Clinicopathological characteristics of the study cohort

	*N*	%
Patients	208	
Age (years)	Median: 66 (IQR: 59–72)	Range: 35–85
FIGO stage		
I	3	1.4
II	11	5.3
III	126	60.6
IV	68	32.7
Type of surgery		
Staging	5	2.4
Primary debulking	55	26.4
Interval debulking	148	71.2
Debulking surgery outcome		
Complete	123	60.6
Optimal	63	31
Incomplete	17	8.4
Number of chemotherapy cycles	Mean and median: 6	Range: 0–8
Median progression-free survival time (months)	17.8	
Median overall survival time (months)	41.5	

^*FIGO*^^, International Federation of Gynecology and Obstetrics^

### Histological subtypes

#### Classification according to criteria of Murakami et al.

**Fig. 2:**
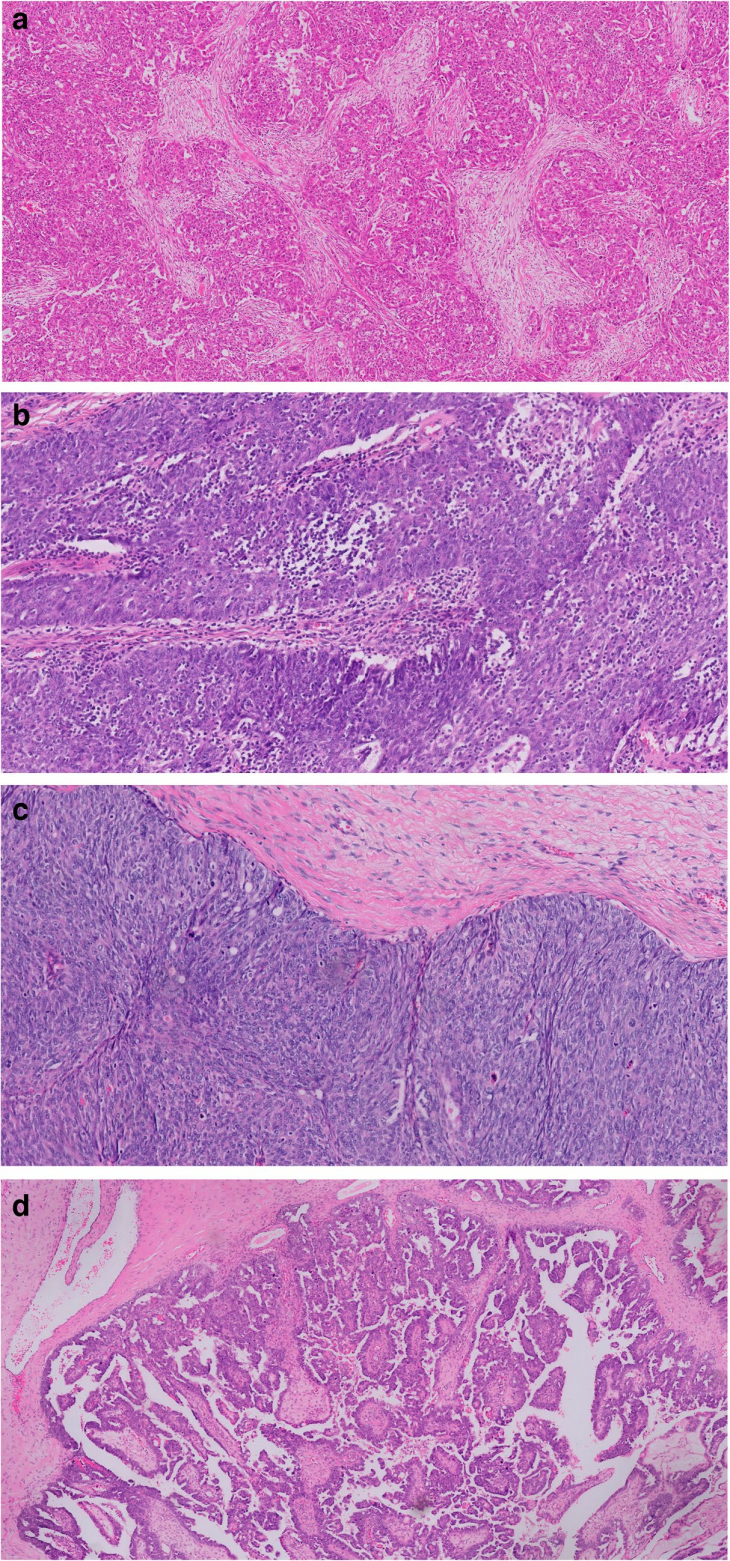
Histopathology of the four subtypes. **a** Example showing the mesenchymal subtype of high-grade serous ovarian carcinoma with infiltrating complex papillary architecture with slit-like spaces (labyrinthine pattern) and small invasive nests, with prominent desmoplastic reaction in > 10% of the tumor area. Original magnification: × 4. **b** Example showing the immunoreactive pattern for both classifications. Murakami et al.: a median of more than 100 surrounding and 50 infiltrating lymphocytes per visual field. Miyagawa et al.: stromal tumor-infiltrating lymphocytes (TILs) in > 25%. Original magnification: × 20. **c** Example showing the solid and proliferative subtype with solid growth without intervening stroma. The surrounding stroma shows minimal desmoplastic reaction. Original magnification: × 10. **d** Example showing the papilloglandular subtype with papillary formations with fibrovascular cores. The surrounding stroma shows minimal desmoplastic reaction. Original magnification: × 4

First, all cases were independently classified by two gynecological pathologists using the method of Murakami et al., with integration of the refined criteria from Miyagawa et al. for non-immunoreactive subtypes (Figs. 1 and 2). After joint evaluation of the discordant cases, 122 patients (58.7%) were classified as mesenchymal, 10 (4.8%) as immunoreactive, 26 (12.5%) as solid and proliferative, and 50 (24%) as papilloglandular.

Both pathologists concurred on the classification in 62.5% of the cases, reflecting a fair level of agreement (*κ* = 0.34, 95% Confidence Interval (CI) 0.23–0.44, *p* < 0.001). For the subtypes as determined by pathologist one, the greatest discrepancies were seen in the immunoreactive and papilloglandular classifications (Table 2). Of the 10 cases categorized as immunoreactive by pathologist one, four (40%) were classified as papilloglandular by pathologist two. Additionally, of the 55 papilloglandular cases classified by pathologist one, 31 (56.4%) were recognized as mesenchymal by pathologist two. Conversely, for the classifications made by pathologist two, the largest discrepancies were observed in the immunoreactive and solid and proliferative subtypes. Specifically, 13 of the 15 (86.7%) cases classified as immunoreactive by pathologist two were classified as mesenchymal by pathologist one, as well as 7 of 27 (25.9%) solid and proliferative cases.

**Table 2:** Pairwise comparison of the subtypes based on the criteria of Murakami et al., classified by pathologists one and two

		Pathologist two				
		Mesenchymal	Immunoreactive	Solid and proliferative	Papilloglandular	Total
Pathologist one	Mesenchymal	**96**	13	7	7	123
	Immunoreactive	2	**2**	2	4	10
	Solid and proliferative	4	0	**13**	3	20
	Papilloglandular	31	0	5	**19**	55
	Total	133	15	27	33	208

In 144 cases (69.2%), the pathologists concurred on the classification of mesenchymal versus non-mesenchymal subtype, with 96 cases identified as mesenchymal and 48 as non-mesenchymal. Conversely, 64 cases were discordant (30.8%). This reflected a fair agreement in differentiating mesenchymal from non-mesenchymal tumors (*κ* = 0.35, 95% CI 0.22–0.48, *p* < 0.001).

#### Classification according to the criteria of Miyagawa et al.

Miyagawa et al. characterized tumors as immunoreactive when lymphocytes infiltrated in ≥ 25% of the stroma (Figs. 1 and 2b). For the other subtypes, the criteria used by Miyagawa et al. were consistent with those described by Murakami et al. Therefore, cases jointly classified as immunoreactive (*n* = 10), solid and proliferative (*n* = 26), and papilloglandular (*n* = 50) based on the criteria of Murakami et al. were independently re-evaluated, followed by joint evaluation in case of discordance. This resulted in the reclassification of six solid and proliferative and seven papilloglandular cases to immunoreactive under the Miyagawa et al. criteria (Table 3). The remaining 73 cases, including all 10 immunoreactive cases, maintained their initial classification. Consequently, according to the classification of Miyagawa et al., 122 cases (58.7%) were mesenchymal, 23 (11%) immunoreactive, 20 (9.6%) solid and proliferative, and 43 (20.7%) papilloglandular.

**Table 3:** Pairwise comparison of the subtypes based on the criteria of Murakami et al. and Miyagawa et al.

		Miyagawa et al.				
		Mesenchymal	Immunoreactive	Solid and proliferative	Papilloglandular	Total
Murakami et al.	Mesenchymal	**122**	0	0	0	122
	Immunoreactive	0	**10**	0	0	10
	Solid and proliferative	0	6	**20**	0	26
	Papilloglandular	0	7	0	**43**	50
	Total	122	23	20	43	208

The pathologists showed concordance in the classification of all 10 cases that were initially jointly identified as immunoreactive, as well as those reclassified from solid and proliferative (*n* = 6) or papilloglandular (*n* = 7) to immunoreactive. Of the 20 solid and proliferative cases and 43 papilloglandular cases that sustained their original classification, there was disagreement about the immunoreactive status in five (7.9%) cases.

### Clinical characteristics of HGSOC histological subtypes

Using the criteria of Murakami et al., patients with the solid and proliferative subtype showed a lower median age (61.5 years) compared to those with the mesenchymal subtype (66 years, *p* = 0.049) and the papilloglandular subtype (69.5 years, *p* = 0.020, Online Resource 1A). In contrast, no differences in median age were observed for the subtypes defined by Miyagawa et al. (Online Resource 1B). There were no differences in FIGO stage, type of surgery, outcome of debulking surgery, and median number of chemotherapy cycles between the subtypes of both classification methods.

#### Progression-free survival

**Fig. 3:**
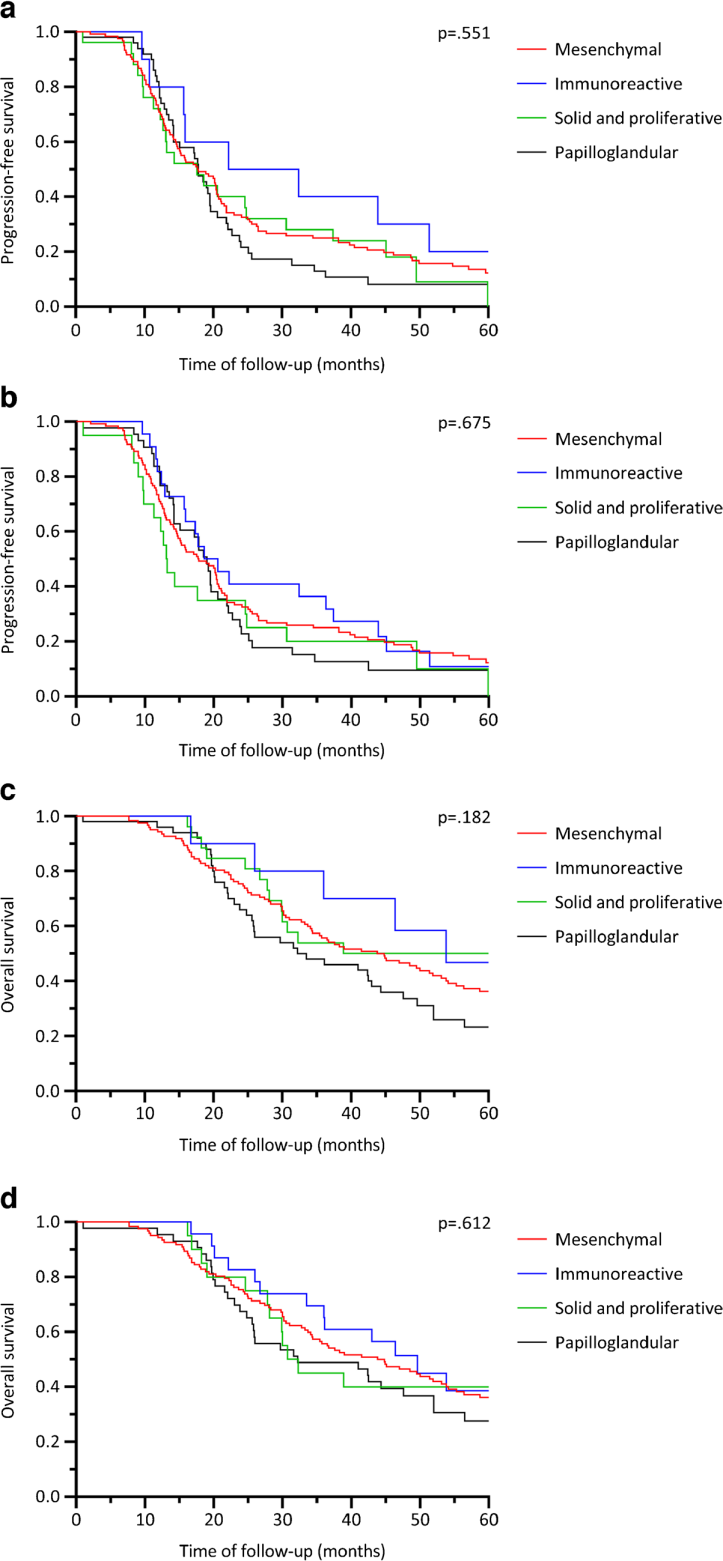
Overall and progression-free survival curves of the subtypes based on the criteria of Murakami et al. and Miyagawa et al. **a** Progression-free survival curves of the subtypes based on the criteria of Murakami et al. **b** Progression-free survival curves of the subtypes based on the criteria of Miyagawa et al. **c** Overall survival curves of the subtypes based on the criteria of Murakami et al. **d** Overall survival curves of the subtypes based on the criteria of Miyagawa et al.

Utilizing the criteria of Murakami et al., the median 5-year PFS of the immunoreactive subtype was 22.2 months. This was followed by 17.8 months for the papilloglandular and mesenchymal subtype, and 17.6 months for the solid and proliferative subtype (Fig. 3a). The 5-year PFS between these subtypes was not distinct (*p* = 0.551). Similarly, no differences in 5-year PFS were observed between the histotypes when applying the criteria of Miyagawa et al. (*p* = 0.675). The papilloglandular subtype had a median PFS of 19 months, the immunoreactive subtype of 18.6 months, the mesenchymal subtype of 17.8 months, and the solid and proliferative subtype of 13.1 months (Fig. 3b).

In a univariate Cox regression analysis, FIGO stage III and IV disease (hazard ratio (HR) 8.82, 95% CI 2.81–27.75, *p* < 0.001) and the presence of residual tumor post-surgery (HR 2.39, 95% CI 1.75–3.25, *p* < 0.001) were found to be statistically associated with a decreased 5-year PFS rate. However, the subtype, irrespective of the classification method employed, showed no impact on PFS (Online Resources 2A and 2B). These findings remained consistent in a subsequent multivariate Cox regression analysis (Online Resources 2A and 2B).

#### Overall survival

Using the criteria proposed by Murakami et al., the immunoreactive HGSOC subtype showed a median OS of 53.8 months, compared with 43.8 months for the mesenchymal subtype, 38.9 months for the solid and proliferative subtype, and 32.3 months for the papilloglandular subtype (Fig. 3c). The 5-year OS rate of these subtypes did not show a difference (*p* = 0.182). Based on the criteria of Miyagawa et al., the median OS of the mesenchymal subtype remained unchanged at 43.8 months. However, the immunoreactive subtype had a median OS of 49.6 months, the papilloglandular subtype of 32.2 months, and the solid and proliferative subtype of 30.7 months (Fig. 3d). Similar to Murakami et al., no difference in 5-year OS between these four subtypes was observed (*p* = 0.612).

A univariate Cox regression analysis showed that FIGO stage III and IV disease (HR 3.23, 95% CI 1.19–8.73, *p* = 0.021) and the presence of residual tumor following surgery (HR 2.63, 95% CI 1.86–3.72, *p* < 0.001) were significantly associated with a reduced OS rate. Conversely, the tumor subtype, regardless of the classification method, did not demonstrate an association with OS (Online Resources 2C and 2D). In a multivariate Cox regression analysis, only the presence of macroscopic residual tumor post-operation was found to be linked to a reduction in OS (Online Resources 2C and 2D).

## Discussion

In this study, we classified 208 HGSOC cases into mesenchymal, immunoreactive, solid and proliferative, or papilloglandular subtypes, based on histopathological criteria established by Murakami et al. and Miyagawa et al. [13, 14]. We observed only a fair interobserver agreement for each method. Importantly, no differences in 5-year progression-free and overall survival rates were observed between the subtypes, irrespective of the classification method employed. Consequently, subtyping HGSOC based on the currently available histopathological classification methods offers no clear advantage in clinical practice.

To make well-informed treatment decisions based on the predicted prognosis and treatment response of the histological subtypes, it is crucial that these subtypes are consistent and reproducible. In the study of Murakami et al. (2016), six observers classified the cases, resulting in an average concordance rate of 74% (range: 61–89%, average Cohen’s *κ* = 0.65, range: 0.46–0.85), a rate they regarded as suboptimal for use in clinical practice [13]. In their subsequent study by Miyagawa et al. (2023), the application of the Murakami et al. criteria yielded a Fleiss’ *κ* of 0.348 between four observers. After refining the definitions of the mesenchymal, solid and proliferative, and papilloglandular subtypes, and adjusting the definition of the immunoreactive subtype due to challenges in observing cellular details on WSI, the coefficient increased to 0.549 [14]. In our study, two specialized gynecological pathologists reached a 62.5% concordance rate (Cohen’s *κ* = 0.34), utilizing the refined criteria of Miyagawa et al., except for the immunoreactive subtype, which was based on the criteria of Murakami et al. It was not possible to calculate Cohen’s *κ* for the cases classified using Miyagawa et al. criteria due to reclassification of tumors jointly classified as immunoreactive, solid and proliferative, or papilloglandular under the Murakami et al. criteria. However, using the immunoreactive definition of Miyagawa et al. likely has a limited influence on the determined Cohen’s *κ*, as only 13 out of 86 cases changed subtype. We argue that this reproducibility rate is not robust enough for clinical utilization in individual patients [16, 17].

The observed low concordance rate in subtype classification may be the underlying reason for the absence of differences in PFS and OS between the subtypes in our study. To minimize misclassification, each case was rigorously reviewed by two specialized gynecological pathologists. However, a possible overrepresentation of the mesenchymal subtype was observed, with 122 of 208 cases (58.7%) being classified as mesenchymal. This contrasts with the 36% in the study of Murakami et al., and 44% in the study of Miyagawa et al. [13, 14]. Cohen’s *κ* for distinguishing mesenchymal versus non-mesenchymal subtype was 0.352 (69.2% concordance rate) in our study. In comparison, the study of Murakami et al. reported a Cohen’s *κ* of 0.70 (85% concordance rate), while the study of Miyagawa et al. reported a Fleiss’ *κ* of 0.703. This difference might contribute to the contrasting survival outcomes for the mesenchymal subtype, as this study could not replicate the lower OS rate for the mesenchymal subtype observed in the study of Murakami et al. Moreover, Murakami et al. reported a higher PFS and OS rate for the immunoreactive subtype compared to the other subtypes. Our study showed a similar, although not statistically significant, trend. A potential explanation for this might be the limited number of cases we classified as immunoreactive using Murakami et al. criteria (*n* = 10, 4.8%). Using the revised definition of Miyagawa et al., this number increased to 23 (11%). However, this did not lead to differences in survival outcomes.

Khashaba et al. (2022) also investigated the correlation between histopathological subtypes of HGSOC and survival outcomes [18]. However, their classification algorithm differed from that of Murakami et al., encompassing variations in both the histopathological subtypes and the criteria used for the classification [13, 18]. Cases featuring a cellular stromal reaction in > 10% of tumor tissue were classified as mesenchymal. All other cases with a mitotic count of > 30/10 high power fields were classified as proliferative. Cases showing a lower mitotic count, but > 20 lymphocytes infiltrating tumor nests per high power field, were categorized as immunoreactive. The remaining cases were categorized as differentiated, and further divided into those with solid endometrioid transitional (SET) or (micro)papillary features [18]. These criteria were derived from different studies and were not compared with the gene expression-based subtypes [13, 19–21]. Therefore, we did not apply this classification method to our cohort. Nevertheless, Khashaba et al. identified the histopathological subtype as a significant factor for PFS (*p* = 0.008) in a univariate analysis, although no significant correlation was found with OS. The mesenchymal subtype demonstrated the shortest median PFS, whereas the differentiated subtype with SET features exhibited the longest median PFS. Additionally, primary debulking surgery, compared to interval debulking surgery, and administration of paclitaxel and carboplatin chemotherapy, compared to other chemotherapeutic agents, also showed correlation with a better PFS (*p* = 0.004 and *p* = 0.001, respectively) [18]. However, multivariate analysis on PFS was not conducted, probably due to the small sample size of the study (*n* = 85). Consequently, the correlation between the histopathological subtype based on the criteria of Khashaba et al. and PFS remains uncertain [18].

Research has also indicated that treatment responses might vary among the subtypes. Specifically, the mesenchymal subtype has been reported to have the most favorable response to taxane-based chemotherapy and potentially to dose-dense chemotherapy as well [11, 13]. Furthermore, bevacizumab is thought to be more efficacious in the mesenchymal and proliferative subtypes [12, 14, 22]. However, since the standard treatment regimen for our patients included carboplatin and paclitaxel, without the addition of bevacizumab, our study could not explore these potential differential responses to therapy.

One of the strengths of our study is that all cases were independently reviewed by two gynecological pathologists. In instances of discordance, a joint review was undertaken, ensuring consistent and accurate subtype classification. While the initial agreement between pathologists was relatively low, consensus was eventually reached for all cases. Furthermore, subtyping was performed by the same two pathologists for both classification systems. This approach effectively eliminated variance between classification systems that might arise from discrepancies in individual pathologist evaluations.

A limitation of our study was that we did not perform RNA sequencing. Consequently, we could not validate whether the subtypes, as identified by the two gynecological pathologists, aligned with those determined by gene expression analysis. In the study of Murakami et al., concordance between the gene expression-based subtypes and histopathological subtypes was examined but limited to a subset of samples (*n* = 59). They observed agreement in approximately 70% of the cases, showing a significant association between the subtypes (*p* < 0.001) [13]. However, given the observed low concordance rate between pathologists in our study, there is a clear need for more straightforward subtyping criteria, particularly for the mesenchymal subtype, as the presence of this subtype may impact the choice of treatment [11–14, 22]. Future investigations should prioritize refining these criteria to improve interobserver consistency, and subsequently examine survival outcomes.

We suggest further exploration of the application of artificial intelligence (AI) or machine learning. The feasibility of histotype classification using machine learning has already been demonstrated [23]. For HGSOC subtyping, AI or machine learning could support the refinement of HGSOC subtype criteria by identifying novel or alternative histopathological features. Moreover, these computational approaches may have the capacity to determine HGSOC subtypes autonomously. However, to facilitate such research, high-quality RNA-sequencing data is necessary, considering the current challenges in achieving adequate reproducibility rates for histopathological subtypes. Once AI or machine learning is refined through training with robust data, it could play an important role in subtyping HGSOC, enhancing diagnostic precision, and informing treatment strategies.

In conclusion, the current histopathological subtypes of HGSOC as defined by Murakami et al. and Miyagawa et al. demonstrate only fair reproducibility. Furthermore, in our cohort of 208 HGSOC patients, no differences in overall and progression-free survival between the subtypes were observed. As such, the implementation of these subtypes in clinical practice remains premature. Further research is warranted to establish criteria that are more straightforward and to investigate their association with survival outcomes.

## Supplementary Information

Below is the link to the electronic supplementary material.

Supplementary file1 (PDF 223 KB)

Supplementary file2 (PDF 355 KB)

## Data Availability

The data that support the findings of this study are available from the corresponding author upon reasonable request.
