# Supplementary material for: Evaluation of the prognostic potential of histopathological subtyping in high-grade serous ovarian carcinoma
Source: Virchows Arch. 2024 Apr 16;486(2):381–91. doi: 10.1007/s00428-024-03807-7 (PMC11876211; doi:10.1007/s00428-024-03807-7)
Supplement: Supplementary file 1 — Supplementary file1 (PDF 223 KB) [file 428_2024_3807_MOESM1_ESM.pdf]

Online Resource 1: Clinical characteristics of the cases based on the subtypes

Online Resource 1A: Clinical characteristics of the cases based on the subtypes defined by Murakami et al.

	Mesenchymal	Immunoreactive	Solid and proliferative	Papilloglandular	p-value
Cases, n (%)	122 (58.7)	10 (4.8)	26 (12.5)	50 (25)	N/A
Age, median (years)	66	69.5	61.5	69.5	*.024*#
FIGO stage, n (%)					.525
I	2 (1.7)	0 (0)	1 (3.8)	0 (0)	
II	6 (4.9)	1 (10)	1 (3.8)	3 (6)	
III	72 (59)	4 (40)	19 (73.1)	31 (62)	
IV	42 (34.4)	5 (50)	5 (19.3)	16 (32)	
Type of surgery, n (%)					.121
Staging	4 (3.3)	0 (0)	1 (3.8)	0 (0)	
Primary debulking	26 (21.3)	2 (20)	12 (46.2)	15 (30)	
Interval debulking	92 (75.4)	8 (80)	13 (50)	35 (70)	
Debulking surgery outcome*					.693
Complete	68 (57.6)	9 (90)	16 (64)	30 (60)	
Optimal	38 (32.2)	1 (10)	8 (32)	16 (32)	
Incomplete	12 (10.2)	0 (0)	1 (4)	4 (8)	
Number of chemotherapy cycles (median)	6	6	6	6	.865

Significant p-values are in italic. *Only patients who underwent primary or interval debulking surgery were analyzed. #Post-hoc testing showed differences between the solid and proliferative and the mesenchymal subtype (adjusted p=.049) and between the solid and proliferative and the papilloglandular subtype (adjusted p=.020). FIGO, International Federation of Gynecology and Obstetrics; N/A, not applicable.

Online Resource 1B: Clinical characteristics of the cases based on the subtypes defined by Miyagawa et al.

	Mesenchymal	Immunoreactive	Solid and proliferative	Papilloglandular	p-value
Cases, n (%)	122 (58.7)	23 (11.1)	20 (9.6)	43 (20.7)	N/A
Age, median (years)	66	70	62	69	.070
FIGO stage, n (%)					.690
I	2 (1.7)	0 (0)	1 (5)	0 (0)	
II	6 (4.9)	2 (8.7)	0 (0)	3 (7)	
III	72 (59)	13 (56.5)	15 (75)	26 (60.4)	
IV	42 (34.4)	8 (34.8)	4 (20)	14 (32.6)	
Type of surgery, n (%)					.306
Staging	4 (3.3)	0 (0)	1 (5)	0 (0)	
Primary debulking	26 (21.3)	9 (39.1)	6 (30)	14 (32.6)	
Interval debulking	92 (75.4)	14 (60.9)	13 (65)	29 (67.4)	
Debulking surgery outcome*					.618
Complete	68 (57.6)	18 (78.3)	10 (52.6)	27 (62.8)	
Optimal	38 (32.2)	4 (17.4)	8 (42.1)	13 (30.2)	
Incomplete	12 (10.2)	1 (4.3)	1 (5.3)	3 (7)	
Number of chemotherapy cycles (median)	6	6	6	6	.689

Significant p-values are in italic. *Only patients who underwent primary or interval debulking surgery were analyzed. FIGO, International Federation of Gynecology and Obstetrics; N/A, not applicable.

Article title	Evaluation of the prognostic potential of histopathological subtyping in high-grade serous ovarian carcinoma
Journal name	Virchows Archiv

Author names	Hein S. Zelisse, Robin A. Hwan, Marc J. van de Vijver, Frederike Dijk, Constantijne H. Mom, Gerrit K.J. Hooijer, Mignon D.J.M. van Gent, Malou L.H. Snijders*
*Corresponding author	Department of Pathology, Cancer Center Amsterdam, Amsterdam UMC, University of Amsterdam, Amsterdam, the Netherlands<br>m.l.h.snijders@amsterdamumc.nl
