# Supplementary material for: Evaluation of the prognostic potential of histopathological subtyping in high-grade serous ovarian carcinoma
Source: Virchows Arch. 2024 Apr 16;486(2):381–91. doi: 10.1007/s00428-024-03807-7 (PMC11876211; doi:10.1007/s00428-024-03807-7)
Supplement: Supplementary file 2 — Supplementary file2 (PDF 355 KB) [file 428_2024_3807_MOESM2_ESM.pdf]

**Online Resource 2:** Univariate and multivariate cox proportional regression analysis

**Online Resource 2A:** Univariate and multivariate cox proportional regression analysis of five-year progression-free survival rate based on subtypes of Murakami et al.

| | Univariate, HR (95% CI) | p-value | Multivariate, HR (95% CI) | p-value |
|---|---|---|---|---|
| Age | | | | |
| <66 | Reference | | | |
| ≥66 | 1.00 (0.75-1.35) | .985 | | |
| FIGO stage | | | | |
| I or II | Reference | | Reference | |
| III or IV | 8.82 (2.81-27.75) | *<.001* | 7.27 (2.30-22.99) | *<.001* |
| Macroscopic residual tumor post-surgery | | | | |
| No | Reference | | Reference | |
| Yes | 2.39 (1.75-3.25) | *<.001* | 2.10 (1.54-2.88) | *<.001* |
| Subtype | | | | |
| Mesenchymal | Reference | | Reference | |
| Immunoreactive | 0.69 (0.34-1.42) | .316 | 0.92 (0.44-1.91) | .822 |
| Papilloglandular | 1.17 (0.82-1.67) | .387 | 1.11 (0.78-1.59) | .557 |
| Solid and proliferative | 1.07 (0.67-1.69) | .779 | 1.10 (0.69-1.75) | .686 |

Significant p-values are in italic. CI, confidence interval; FIGO, International Federation of Gynecology and Obstetrics.

**Online Resource 2B:** Univariate and multivariate cox proportional regression analysis of five-year progression-free survival rate based on subtypes of Miyagawa et al.

| | Univariate, HR (95% CI) | p-value | Multivariate, HR (95% CI) | p-value |
|---|---|---|---|---|
| Age | | | | |
| <66 | Reference | | | |
| ≥66 | 1.00 (0.75-1.35) | .985 | | |
| FIGO stage | | | | |
| I or II | Reference | | Reference | |
| III or IV | 8.82 (2.81-27.75) | *<.001* | 7.30 (2.31-23.08) | *<.001* |
| Macroscopic residual tumor post-surgery | | | | |
| No | Reference | | Reference | |
| Yes | 2.39 (1.75-3.25) | *<.001* | 2.12 (1.55-2.90) | *<.001* |
| Subtype | | | | |
| Mesenchymal | Reference | | Reference | |
| Immunoreactive | 0.87 (0.53-1.42) | .579 | 1.04 (0.63-1.71) | .894 |
| Papilloglandular | 1.10 (0.75-1.60) | .636 | 1.05 (0.72-1.53) | .821 |
| Solid and proliferative | 1.26 (0.77-2.09) | .360 | 1.24 (0.75-2.06) | .395 |

Significant p-values are in italic. CI, confidence interval; FIGO, International Federation of Gynecology and Obstetrics.

**Online Resource 2C:** Univariate and multivariate cox proportional regression analysis of five-year overall survival rate based on subtypes of Murakami et al.

| | Univariate, HR (95% CI) | p-value | Multivariate, HR (95% CI) | p-value |
|---|---|---|---|---|
| Age | | | | |
| <66 | Reference | | | |
| ≥66 | 1.11 (0.79-1.57) | .549 | | |
| FIGO stage | | | | |
| I or II | Reference | | Reference | |
| III or IV | 3.23 (1.19-8.73) | *.021* | 2.42 (0.89-6.62) | .084 |
| Macroscopic residual tumor post-surgery | | | | |
| No | Reference | | Reference | |
| Yes | 2.63 (1.86-3.72) | *<.001* | 2.45 (1.72-3.50) | *<.001* |
| Subtype | | | | |
| Mesenchymal | Reference | | Reference | |
| Immunoreactive | 0.67 (0.27-1.65) | .382 | 0.97 (0.38-2.43) | .941 |
| Papilloglandular | 1.37 (0.92-2.03) | .117 | 1.31 (0.88-1.95) | .177 |
| Solid and proliferative | 0.80 (0.44-1.44) | .456 | 0.81 (0.45-1.45) | .473 |

Significant p-values are in italic. CI, confidence interval; FIGO, International Federation of Gynecology and Obstetrics.

**Online Resource 2D:** Univariate and multivariate cox proportional regression analysis of five-year overall survival rate based on subtypes of Miyagawa et al.

| | Univariate, HR (95% CI) | p-value | Multivariate, HR (95% CI) | p-value |
|---|---|---|---|---|
| Age | | | | |
| <66 | Reference | | | |
| ≥66 | 1.11 (0.79-1.57) | .549 | | |
| FIGO stage | | | | |
| I or II | Reference | | Reference | |
| III or IV | 3.23 (1.19-8.73) | *.021* | 2.45 (0.90-6.70) | .080 |
| Macroscopic residual tumor post-surgery | | | | |
| No | Reference | | Reference | |
| Yes | 2.63 (1.86-3.72) | *<.001* | 2.50 (1.75-3.56) | *<.001* |
| Subtype | | | | |
| Mesenchymal | Reference | | Reference | |
| Immunoreactive | 0.85 (0.47-1.52) | .574 | 1.05 (0.58-1.91) | .865 |
| Papilloglandular | 1.26 (0.83-1.93) | .282 | 1.25 (0.82-1.90) | .309 |
| Solid and proliferative | 1.03 (0.56-1.90) | .918 | 0.92 (0.50-1.70) | .786 |

Significant p-values are in italic. CI, confidence interval; FIGO, International Federation of Gynecology and Obstetrics.

| | |
|---|---|
| Article title | Evaluation of the prognostic potential of histopathological subtyping in high-grade serous ovarian carcinoma |
| Journal name | Virchows Archiv |
| Author names | Hein S. Zelisse, Robin A. Hwan, Marc J. van de Vijver, Frederike Dijk, Constantijne H. Mom, Gerrit K.J. Hooijer, Mignon D.J.M. van Gent, Malou L.H. Snijders* |
| *Corresponding author | Department of Pathology, Cancer Center Amsterdam, Amsterdam UMC, University of Amsterdam, Amsterdam, the Netherlands<br>m.l.h.snijders@amsterdamumc.nl |
